# Sporadic Creutzfeldt-Jakob Disease: Prion Pathology in Medulla Oblongata—Possible Routes of Infection and Host Susceptibility

**DOI:** 10.1155/2015/396791

**Published:** 2015-09-17

**Authors:** Diego Iacono, Sergio Ferrari, Matteo Gelati, Gianluigi Zanusso, Sara Mariotto, Salvatore Monaco

**Affiliations:** ^1^Neuropathology Research, Biomedical Research Institute of New Jersey, Cedar Knolls, NJ 07960, USA; ^2^Department of Pathology, Johns Hopkins University School of Medicine, Baltimore, MD 21218, USA; ^3^Department of Neurological and Movement Sciences, Neurology and Neuropathology Unit, University of Verona, Policlinico G.B. Rossi, Piazzale L.A. Scuro 10, 37134 Verona, Italy

## Abstract

Sporadic Creutzfeldt-Jakob disease (sCJD), the most frequent human prion disorder, is characterized by remarkable phenotypic variability, which is influenced by the conformation of the pathologic prion protein and the methionine/valine polymorphic codon 129 of the prion protein gene. While the etiology of sCJD remains unknown, it has been hypothesized that environmental exposure to prions might occur through conjunctival/mucosal contact, oral ingestion, inhalation, or simultaneous involvement of the olfactory and enteric systems. We studied 21 subjects with definite sCJD to assess neuropathological involvement of the dorsal motor nucleus of the vagus and other medullary nuclei and to evaluate possible associations with codon 129 genotype and prion protein conformation. The present data show that prion protein deposition was detected in medullary nuclei of distinct sCJD subtypes, either valine homozygous or heterozygous at codon 129. These findings suggest that an “environmental exposure” might occur, supporting the hypothesis that external sources of contamination could contribute to sCJD in susceptible hosts. Furthermore, these novel data could shed the light on possible causes of sCJD through a “triple match” hypothesis that identify environmental exposure, host genotype, and direct exposure of specific anatomical regions as possible pathogenetic factors.

## 1. Introduction

Prion diseases, or transmissible spongiform encephalopathies (TSEs), are mammalian neurodegenerative disorders that occur as sporadic, inherited, or iatrogenic forms [1]. The crucial pathogenetic event in TSEs is the conformational conversion of the cellular prion protein, or PrP^C^, into PrP^Sc^, a self-propagating detergent-insoluble and protease-resistant isoform [2]. In different prion strains, PrP^Sc^ shows distinct sites of endogenous or exogenous proteolysis generating a core fragment named PrP27-30 [3]. Sporadic Creutzfeldt-Jakob disease (sCJD), the most common TSE, accounts for about 85% of all human prion diseases and is characterized by the absence of mutations in the prion protein gene (*PRNP*) and by lack of epidemiological evidence for iatrogenic, dietary, or contact exposure to human or animal TSEs agents. Six different molecular subtypes of sCJD are recognized, based on the genotype at codon 129 of the* PRNP*, a site of common M/V polymorphism, and two distinct conformers of the pathological prion protein [4]. To date, the etiology of sCJD remains unknown, and many investigations have been devoted to assess whether the disease may follow endogenous generation of prions or exogenous infection. If sCJD occurs spontaneously, the disease might be initiated by somatic mutations in* PRNP* at sites of ongoing neurogenesis, such as the olfactory mucosa and the olfactory bulb, or by stochastic misfolding of the PrP^C^ in neural tissues. Conversely, if exogenous infection is the cause, the gastrointestinal tract (GIT), conjunctival or mucous membranes, and the olfactory system are the routes of entry to be taken into consideration. Naturally occurring mammalian prion infections due to foodborne contamination include Kuru, BSE, and vCJD [5]. However, in these disorders the route of prions from the GIT to the central nervous system remains largely unknown. Experimental models show that after intragastric infection neuroinvasion sequentially occurs via Peyer's patches and the enteric nervous system (ENS) [6, 7]. Neuroinvasion may spread from the ENS through the splanchnic nerves to the intermediolateral columns of the spinal cord or to the vagus nerve to reach the nodose ganglion and the dorsal motor nucleus vagus (DMNV) [8]. It is possible, however, that distinct prion strains, especially lymphotropic agents, use alternative routes for the neuroinvasion [9].

Recently, a so-called “dual hit” hypothesis has been proposed for Parkinson's disease (PD); this theory postulates a simultaneous involvement of the olfactory system and the DMNV, following aerosolic exposure to an unidentified pathogen which spreads to the ENS after swallowing [10]. Intriguingly, the dual hit hypothesis seems to fit well with a more general theory that could include prions.

Here we investigate the involvement of the DMNV and other medullary nuclei in 21 consecutive definite sCJD cases with different molecular subtypes.

## 2. Material and Methods

### 2.1. Demographic and Clinical Data

Twenty-one consecutive unrelated subjects (10 males and 11 females) with definite sCJD were examined in the study. Clinical data and relevant hospital records, including MRI, EEG, and CSF assessment [11], were obtained from the treating physicians in Neurological and Geriatric Units of Veneto (17), Trentino-Alto Adige (1), Friuli-Venezia Giulia (2), and Lombardia (1) in Northern Italy. All data were obtained after the permission of the local competent legal and ethical authorities. Brains obtained at autopsy were collected during the period 1998–2003 at Neuropathology Unit, University of Verona, Italy.

None of these sCJD cases had a history of possible external or iatrogenic exposure. The duration of disease was calculated from the onset of the first symptoms until death. Neurological signs were classified as occurring at “clinical presentation” when observed within the first two weeks of disease. All main demographic and clinical data are summarized in Table 1.

### 2.2. Genetic Studies and PrP^Sc^ Typing

The sequencing of the coding region of* PRNP* was performed in all patients. None presented a pathogenetic mutation of* PRNP*. DNA was extracted from frozen brain tissues. Search for* PRNP* mutations and M/V polymorphism at codon 129 was performed as previously described [12].

Typing of PrP^Sc^ was done as previously described and classified as type 1 and type 2, according to Parchi et al. [4].

### 2.3. Tissue Sampling and Immunohistochemistry

In all cases, the medulla oblongata was sampled at two levels: at the caudal portion of the inferior olive and midway the inferior olive. Eight *μ*m thick paraffin sections were deparaffinized, rehydrated, treated with 98% formic acid for 1 h at room temperature, and then autoclaved with 1.5 mM HCL for 10 minutes at 121°C. After rinsing with distilled H_2_O and phosphate buffered saline (PBS) sections were saturated with 10% goat serum in PBS for 20 minutes, washed in PBS, and incubated overnight at 4°C with anti-PrP monoclonal antibody (3F4, Dako, Denmark) diluted at 1 : 2000 in PBS. Preparations were then incubated with a biotinylated goat anti-mouse secondary antibody 1 : 2000 for 60 minutes at room temperature and finally with the avidin-biotin-peroxidase complex (Vectastain ABC-Elite kit, Vector Laboratories). Diaminobenzidine tetrachloride was used to visualize the immunoreactivity. Subsequently, sections were counterstained with hematoxylin and then dehydrated. The presence of gliosis was assessed with polyclonal rabbit anti-GFAP antibody (Dako, Denmark). Additionally, all the sections were stained with hematoxylin and eosin (H&E). Semiquantitative assessment of PrP^Sc^ deposition, spongiosis, and gliosis was rated as follows: negative (−), mild (±), moderate (+), and severe (++) by three independent neuropathologists.

**Figure 1:**
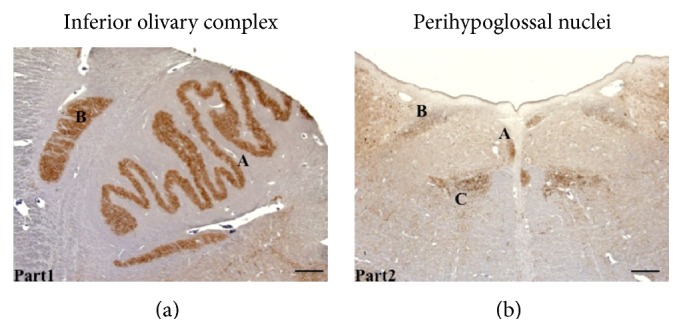
(a) Anti-PrP^Sc^ staining of the inferior olivary complex (IOC): inferior olivary nucleus (A) and accessory olivary nucleus (B) in patient number 14; (b) shows perihypoglossal nuclei: nucleus praepositus (A), nucleus intercalatus (B), and the nucleus of Roller (C), stained with anti-PrP antibody in the same case (bar = 200 *μ*m).

The perihypoglossal nuclei (PHN, including nucleus praepositus, nucleus intercalatus, and the nucleus of Roller) and the inferior olivary complex (IOC, including the medial and dorsal accessory olive and the inferior olive) were considered as whole groups (Figure 1).

### 2.4. Statistics

Statistic columns were calculated to obtain the mean age at death, the mean duration of disease, and the percentages of PrP^Sc^ positivity, respectively, in DMNV, HN, PHN, and IOC of all 21 subjects.

Statistical analyses were also performed clustering the whole sample of subjects in two main groups: patients without V (Group 1) and patients with V (Group 2), that is, subjects with at least one V allele in the genotype.

Parametric analyses, as Student's* t*-tests and one-way analysis of variance, were performed to compare the mean age at death (years) and the mean duration of disease (months) between Group 1 and Group 2. Nonparametric analyses, as Pearson's *χ* ^2^ tests, were used to verify possible significant associations between the PrP^Sc^ positivity in DMNV, HN, PHN, and IOC and Group 1 or Group 2. Pearson's *χ* ^2^ tests were also used to test possible associations between prion positivity of DMNV, HN, PHN, IOC, and, respectively, PrP^Sc^ type 1 and PrP^Sc^ type 2, independently of the M/V genotype.

## 3. Results

All main clinical and neuropathological data are summarized, respectively, in Tables 1 and 2.

The column statistics showed a mean age equal to 64.7 ± 1.5SE years (range: 48–79). The average disease duration was 9.8 ± 2.1SE months (range: 3–48). The percentage of PrP^Sc^ positivity in each nucleus was as follows: 47.6% (10 out of 21 subjects) for DMNV, 33.3% (7 out of 21 subjects) for HN, 52.3% (11 out of 21) for PHN, and 57.1% (12 out of 21) for IOC. There was no statistical difference in terms of percentages PrP^Sc^ positivity across all nuclei.

**Figure 2:**
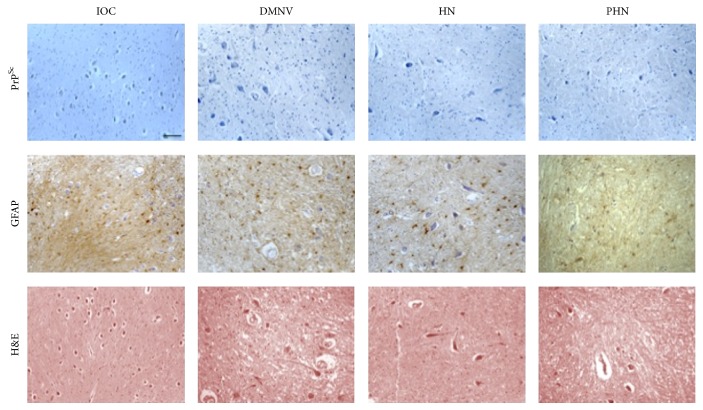
MM1 PrP^Sc^-negative patients. This figure shows the IOC, DMNV, HN, and PHN stained for PrP^Sc^, GFAP, and H&E in MM1 number 2 case. PrP^Sc^ immunostaining results negative in examined nuclei (bar = 50 *μ*m).

**Figure 3:**
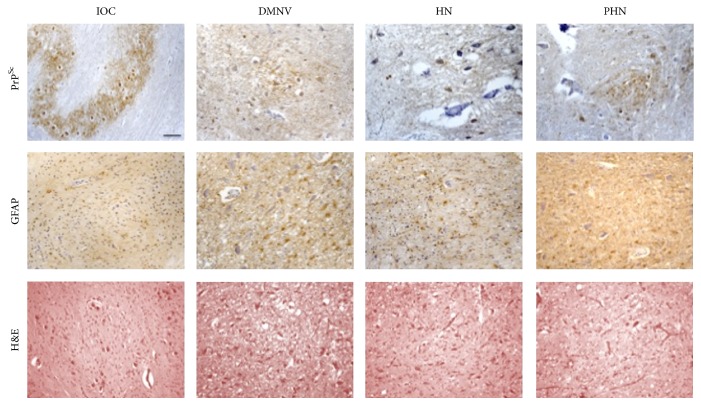
MM1 PrP^Sc^-positive patients. The figure shows the synaptic pattern of PrP^Sc^ deposits in IOC, DMNV, HN, and PHN in MM1 number 3 patient. GFAP and H&E staining are also reported (bar = 50 *μ*m).

**Figure 4:**
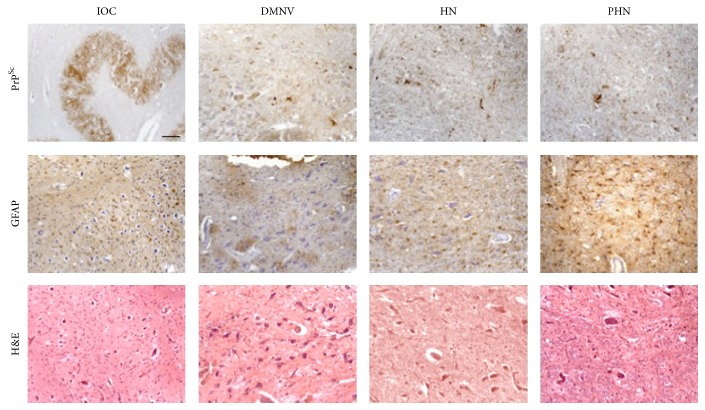
MV2 PrP^Sc^-positive patients. The figure shows a prevalent synaptic pattern of PrP^Sc^ deposits in the IOC, DMNV, HN, and PHN in MV2 number 16 patient. Also granular and plaque-like deposits are evident. GFAP and H&E staining are reported below (bar = 50 *μ*m).

**Figure 5:**
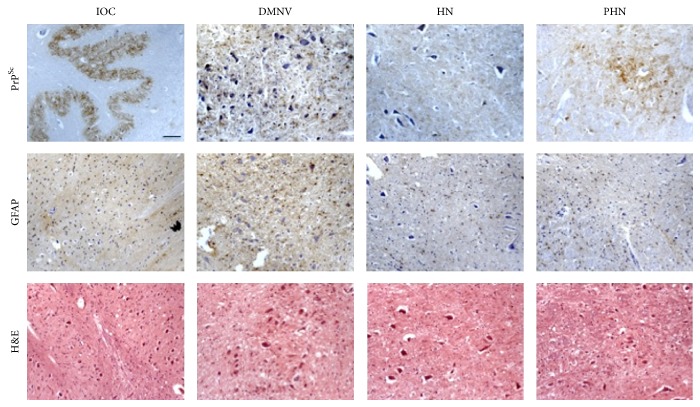
VV2 PrP^Sc^-positive patients. The figure shows PrP^Sc^, GFAP, and H&E staining in IOC, DMNV, HN, and PHN in VV2 number 21 patient. The synaptic and granular patterns of PrP^Sc^ deposits are evident (bar = 50 *μ*m).

In terms of standard neuropathological assessment for CJD (gliosis and spongiosis), all examined cases, independently of the M/V genotype or PrP^Sc^ type, showed low level or absence of spongiosis in the medulla oblongata. In particular no spongiosis was present in IOC, and variable degrees of gliotic reaction were observed in all other nuclei (Figures 2, 3, 4, and 5). There was no linear correlation between PrP^Sc^ positivity, gliotic reaction, and spongiosis across all nuclei. As for the prevalent histological type of PrP^Sc^ deposition, synaptic and granular deposits had a major frequency compared to the other pattern.

The statistical analyses did not show significant differences between Group 1 and Group 2, respectively, subjects with and without V, in terms of mean age at death (Group 1: 64.3 ± 1.6SE, Group 2: 65.3 ± 2.8SE; *P* = 0.75), and duration of disease (Group 1: 7.5 ± 1.5SE; Group 2: 12.7 ± 4.6SE; *P* = 0.24; *F* = 6.7).

Strikingly, the analysis showed significant association (*P* < 0.0001) between Group 2 and PrP^Sc^ positivity in DMNV, HN, PHN, and IOC compared to Group 1.

However, it appears that the polymorphic codon V is associated with prion positivity of all nuclei more than PrP^Sc^ type 2. In fact, the presence of PrP^Sc^ type 2 appeared neither sufficient nor necessary to determine prion protein deposition as demonstrated by MM2 cases (case 11 and case 12). As confirmation, case 13 showed that the presence of V in the absence of PrP^Sc^ type 2 was sufficient for determinate prion positivity in all examined nuclei.

## 4. Discussion

Previous investigations on sCJD pathology showed that the brainstem was essentially spared from the disease process in most of the examined cases [13]. In addition, PrP^Sc^ was mostly absent in series of investigated cases, with a single exception [14]. However, some evidences of variable spongiotic changes of the quadrigeminal plate, substantia nigra, and pontine nuclei have been rarely reported [15].

In the present study we show that PrP^Sc^ deposition occurs in the medulla of subjects affected by sCJD. In particular, PrP^Sc^ deposition was preferentially observed in sCJD subjects with MV2 and VV2 molecular subtypes. Among subjects with MM1/MV1 subtype, 4 cases out of 11 showed PrP^Sc^ deposition in distinct medullary nuclei, although the majority of cases were negative (Table 2). The assessment of the pathological score (gliosis and spongiosis) did not show major differences between cases with or without PrP^Sc^ deposition, since only low-to-mild and unspecific changes could be observed in almost all investigated medullary locations, either at the level of medullary nuclei or in the white matter. This finding confirms previous observations showing that deposition of PrP^Sc^ in the brainstem is not always accompanied by the sequential appearance of spongiform changes, neuronal loss, and astrocytosis, as observed in other CNS regions. These observations suggest also the relative resistance of the brainstem to the pathological changes induced by abnormal PrP^Sc^. This peculiarity may also account for the lack of distinct clinical features, that is, focal midbrain signs, even in cases showing prevailing involvement of medullary nuclei. Differences in the pathological profiles were not accounted by distinct clinical features or disease duration. More importantly, the ongoing findings suggest that specific brainstem structures may represent a possible site for the rostral spreading of prions.

It could be hypothesized that only the intimate molecular interaction between PrP^Sc^ type and a specific genotype could determine the risk of brainstem prion deposition that triggers a transsynaptic mechanism of pathologic spreading.

Our study is partially supported by a previous investigation on a large series of definite sCJD cases, where subtypes MM1 (the majority), MV1, and VV1 were characterized by sparing of the brainstem and almost complete absence of the pathological prion protein [16]. On the other side, the MM2-thalamic phenotype showed moderate/severe pathology in periaqueductal medullary gray and inferior olives. In MV2 and VV2 subtypes a moderate-to-severe pathological score, quantified according to the combined extent of spongiosis, gliosis, and neuronal loss, was observed in the substantia nigra, periaqueductal gray, locus coeruleus, and periventricular medullary gray and inferior olives.

In comparison with these data, the lack of medullary involvement in MM2-cortical subtype and the positivity of MV1 subtype reported in our analysis may support the dominant effect of synonymous or nonsynonymous valine expression at* PRNP* codon 129 over the agent strain in determining the phenotype. However, in previous studies we have demonstrated that the combination of the PrP^Sc^ core fragment and truncated fragments shows two different signatures between type 2 PrP^Sc^ detected in MM subjects of the cortical subtype and that found in MV and VV subjects [3].

In alternative, as based on the M/V polymorphism at the codon 129 of the* PRNP* gene, it is possible that the presence of V could be,* per se*, an important genotypic factor associated with PrP^Sc^ deposition in specific brainstem nuclei. The present data are in line with previous observations that V homozygosity favors the occurrence of iatrogenic CJD (iCJD) when exposure to external contaminated material occurs [17–19] and could be also a determinant of sCJD by prion-contaminated soil [20] or by mucosal/olfactory contact with the environment [21]. Other than representing a risk factor for peripheral selection of PrP^Sc^, V expression could be a major determinant of the selective vulnerability of brainstem nuclei DMNV or HN that are directly connected with the external environment and could be sites of entrance, or transmission, of an exogenic neurotrophic/prion toxic factor. These regions are indeed in direct contact with possible “hostile” outer environments from where both olfactory/oropharyngeal/gastroenteric systems could be “contaminated.”

It has been already hypothesized that environmental exposure to prions may theoretically occur through conjunctival/mucosal contact, olfactory inhalation, and oral ingestion [21–25]. Neuropathological studies have shown that Kuru is characterized by significant brainstem pathology, with severe spongiosis and gliosis of periaqueductal grey and quadrigeminal plate, and less intense changes in the basis pontis, central tegmental area, and inferior olivary nuclei, accompanied by synaptic-type PrP^Sc^ deposition. On the contrary, in vCJD spongiosis, gliosis, and dense synaptic PrP^Sc^ deposition mainly occur within the superior colliculi, substantia nigra, and pontine nuclei. Intriguingly, neuronal loss, spongiosis, and dense PrP^Sc^ deposition are also found within the dorsal motor vagal nuclei and the inferior olivary nuclei [26, 27]. These findings are in keeping with experimental evidence showing that, in sheep orally inoculated with BSE, PrP^Sc^ is first detected in the dorsal motor nucleus of the vagus, hence suggesting a major role of the vagus nerve circuit in neuroinvasion [28]. In addition, these novel findings could match with the “dual hit” hypothesis formulated by Braak for PD [10]. A double prion attack could be also possible in sCJD. The anterograde-way, via olfactory system, and the retrograde-way, via GIT and preganglionic vagal fibers, could be the main anatomical substrates through which the prions could spread out to the rest of the nervous system. Our results suggest that this possibility is real one in susceptible hosts: the V carriers. Moreover, as suggested by Braak, a direct access to the medulla via the viscerosensory fibers of the vagus in the pharynx or trigeminal nerve is not compatible with the sparing of the solitary tract. We agree on this point since we never observed in our sCJD series prion positivity in the solitary tract, not in MM1/MV1, MM2, MV2, or VV2 cases. From this, we speculate that a larger aetiopathogenetic hypothesis could be formulated for sCJD at least (and probably for PD also), named “*triple match,*” including the toxic environmental exposure, the host genotype, and the direct contact with “hot” anatomical regions (i.e., olfactory fibers, vagal fibers, and hypoglossal fibers).

As final finding, it is important to underline that the definition of the lesion profiles, including medulla oblongata, and the regional distribution of the pathological prion protein in brains of subjects with sCJD is important for evaluating cases with suspected environmental origin such as for the epidemiological surveillance of the disorder. In conclusion our data suggest a possible association between the presence of V at codon 129 and deposits of PrP^Sc^ in the nuclei of the medulla oblongata related to the gastrointestinal and olfactory system and highlight a probable distinct mechanism of PrP^Sc^ spreading in patients with sCJD and V at codon 129 of* PRNP* gene.

## Figures and Tables

**Table 1:** Main demographic and clinical data of all subjects in study. Group 2 is the second part of the table.

Case number	sCJD molecular type	Age at death/gender	Duration of disease (months)	Clinical signs at onset	Clinical signs at late clinical evaluation
1	MM1	70/f	3	Confusion	Myoclonus, ataxia, gait disorders, paraphasia, and coma
2	MM1	70/f	4	Cognitive impairment	Progressive cognitive impairment
3	MM1	63/m	6	Irritability, restlessness	Myoclonus, ataxia, motor perseverations, amnesia, loss of spontaneous movement, and aphasia
4	MM1	52/m	3	Amnesia, diplopia, and involuntary movements	Myoclonus, involuntary movements, bradikinesia, cognitive impairment, visual hallucinations, and aphasia
5	MM1	70/f	8	Confusion, hallucinations	Myoclonus, ataxia, progressive dementia, and coma
6	MM1	65/m	3	Confusion, gait disorders	Myoclonus, plastic hypertonia, ataxia, aphasia, and progressive cognitive impairment
7	MM1	71/f	16	Objective vertigo	Myoclonus evoked by auditory and tactile stimuli, ataxia hallucinations, paresthesias, diplopia, alien hand, and dementia
8	MM1	62/f	11	Ataxia, speech disorder	Myoclonus, behavioral changes (aggression), confusion, dysphagia, and spasticity
9	MM1	60/m	2	Confusion	Myoclonus, insomnia, visual hallucinations, apraxia, and cognitive impairment
10	MM1	69/m	6	Visual disorders	Myoclonus, ataxia, and cognitive impairment
11	MM2	61/f	11	Amnesia, impaired attention, and disorientation in time and place	Myoclonus, rigidity, alteration production and comprehension of the speech, agitation, altered sleep wake rhythm, and behavioral disorders
12	MM2	59/m	18	Depression	Amnesia, reduced ideational, ataxia insomnia, headache, cognitive impairment, amnesia, and reduced ideation

13	MV1	66/m	17	Aphasia	Tremor, involuntary movements, dysarthria, comprehension deficit, and akinetic mutism
14	MV2	79/m	6	Gait disorders, drowsiness	Myoclonus, motor slowing, ataxia, and impaired eye movements upwards
15	MV2	61/f	11	Ataxia	Myoclonus, ataxia dementia, and aphasia
16	MV2	48/m	48	Irritability, apathy, delusions, and paranoia	Rigidity, hypokinesia, visuospatial and memory disorders, and cognitive impairment
17	VV2	71/f	5	Dizziness	Myoclonus, photophobia, amnesia, confusion, cognitive impairment, drowsiness, and anxiety
18	VV2	64/f	13	Ataxia, cognitive impairment	Involuntary movements, ataxia depression, cognitive impairment, and delirium
19	VV2	62/f	3	Ataxia	Myoclonus, visual hallucinations, and gaze deviation to the left
20	VV2	68/f	6	Balance disorders	Bradikinesia, myoclonus, ataxia, disorders of attention, difficulty writing, and depression
21	VV2	69/f	6	Cognitive decline, myoclonus	Akinetic mutism

**Table 2:** Quantitative neuropathological analysis of spongiosis, gliosis, and PrP^Sc^ deposits in all the examined nuclei according to the different genotypes. The main pattern of PrP^Sc^ deposits is also reported. Group 2 is the second part of the table.

Case number	sCJD molecular type	DMNV			HN			PHN			IOC		
		S	G	PrP^Sc^	S	G	PrP^Sc^	S	G	PrP^Sc^	S	G	PrP^Sc^
1	MM1	±	±	−	±	±	−	−	±	−	−	±	−
2	MM1	−	±	−	−	−	−	−	−	−	−	±	−
3	MM1	+	+	+ (syn.)	±	+	+ (syn.)	−	±	+ (syn.)	−	±	+ (syn.)
4	MM1	±	±	−	+	+	−	+	+	−	−	−	−
5	MM1	±	−	−	−	−	−	−	−	+ (syn.)	−	−	+ (syn.)
6	MM1	−	−	−	−	−	−	−	−	−	−	−	−
7	MM1	±	±	−	±	±	−	±	±	−	−	±	+ (syn.)
8	MM1	+	+	−	±	±	−	−	−	−	−	−	−
9	MM1	+	++	−	+	±	−	−	−	−	−	−	−
10	MM1	±	−	−	+	±	−	−	±	−	−	−	−
11	MM2	+	±	−	−	−	−	−	−	−	−	−	−
12	MM2	±	±	−	±	±	−	−	−	−	−	−	−

13	MV1	++	++	±	+	+	−	±	±	± (syn.)	−	±	+ (syn.)
14	MV2	+	+	+ (syn.)	±	+	+ (syn.)	±	+	+ (syn.)	−	±	+ (syn.)
15	MV2	±	±	+ (syn.)	±	±	+ (syn.)	±	±	+ (syn.)	−	+	+ (syn.)
16	MV2	−	−	+ (syn.)	+	±	+ (gran., pl.-like)	+	+	+ (syn.)	−	±	+ (syn.)
17	VV2	±	±	+ (syn., periaxo.)	±	±	+ (syn., periaxo.)	±	±	+ (syn., periaxo.)	−	±	+ (syn., periaxo.)
18	VV2	±	++	±	±	+	±	+	+	+	−	+	+
19	VV2	±	±	+	−	−	±	−	−	+	−	±	+
20	VV2	±	±	+ (syn.)	−	±	−	−	±	+ (syn.)	−	±	+ (syn.)
21	VV2	±	+	+ (syn., gran., periaxo.)	±	+	−	±	+	+ (syn., gran., periaxo.)	−	±	+ (syn.)

DMNV: dorsal motor nucleus of the vagus; HN: hypoglossal nucleus; PHN: perihypoglossal nuclei; IOC: inferior olivary complex; S: spongiosis; G: gliosis; PrP^Sc^: prion protein;syn.: synaptic; gran.: granular; periaxo.: periaxonal; pl.-like: plaque-like; −: negative; ±: mild; +: moderate; ++: severe.
